# Osteoporosis in Systemic Autoinflammatory Diseases: A Case-Control Study

**DOI:** 10.3389/fendo.2019.00636

**Published:** 2019-09-18

**Authors:** Sara Bindoli, Giulio Franceschet, Paola Galozzi, Martina Zaninotto, Valentina Camozzi, Paolo Sfriso

**Affiliations:** ^1^Rheumatology Unit, Department of Medicine DIMED, University of Padova, Padova, Italy; ^2^Endocrinology Unit, Department of Medicine DIMED, University of Padova, Padova, Italy; ^3^Department of Laboratory Medicine, University-Hospital of Padova, Padova, Italy

**Keywords:** autoinflammatory diseases, serum amyloid A (SAA), osteoprotegerin, RANKL, osteoporosis

## Abstract

**Objective:** To assess if patients affected by systemic autoinflammatory diseases (SAIDs) present an increased risk of osteoporosis (OP).

**Methods:** Forty adults patients referred to the Rheumatology Unit of Padova University Hospital affected by Familial Mediterranean Fever (FMF), TNF-Receptor Associated Periodic Syndrome (TRAPS), and Mevalonate Kinase Deficiency (MKD) and 40 healthy subjects were enrolled. Blood and urine samples were collected in order to define phosphocalcic metabolism, including Receptor activator of nuclear factor kappa-B ligand (RANKL) and osteoprotegerin (OPG), and among inflammatory markers serum amyloid A (SAA). Femur and lumbar dual-energy X-ray absorptiometry (DXA) scans were performed and Trabecular Bone Score (TBS) was calculated on DXA lumbar images.

**Results:** We did not observe a statistically significant difference between Bone Mineral Density (BMD) and TBS of patients compared to controls. Also, the values of phosphocalcic metabolites in patients did not statistically differ from those in controls. However, SAA and OPG levels were significantly higher in patients compared to healthy subjects (*p* = 0.0244 and *p* = 0.0064, respectively).

**Conclusion:** Patients of our cohort affected by FMF, TRAPS, and MKD do not present an increased risk of OP compared to the healthy controls. TBS and BMD are similar between the two groups underlining a preserved bone quality in patients. High OPG levels could suggest a protective role and a bone re-balancing action in response to an inflammatory background. Finally, it should be taken into account a modulatory role played by a pro-inflammatory cytokine such as SAA on bone homeostasis.

## Introduction

Systemic autoinflammatory diseases (SAIDs) represent a group of disorders of the innate immune system characterized by episodes of systemic inflammation such as recurrent fever attacks with polyserositis and skin, musculo-skeletal, and articular involvement. Raised levels of erythrocyte sedimentation rate (ESR), C-reactive protein (CRP), and serum amyloid A (SAA) often occur (1). In the present work we included adult patients affected by hereditary periodic fevers, in particular patients with Familial Mediterranean Fever (FMF), TNF Receptor Associated Periodic Syndrome (TRAPS), and Mevalonate Kinase Deficiency (MKD) (2). Different studies show that chronic inflammation, perpetuated by pro-inflammatory cytokines, may exert a role on bone metabolism, eventually leading to osteoporosis (OP) onset (3–5). However, the prevalence of OP in the context of autoinflammatory diseases remains partially unknown. Therefore, the aims of our study are focused on the assessment of bone metabolism in patients affected by SAIDs compared to healthy subjects, and on the relationship between bone turnover markers (BTM) (receptor activator of nuclear factor kappa-B ligand, RANKL, and osteoprotegerin, OPG), and inflammatory marker (SAA).

## Subjects and Methods

Forty adult patients referred to the Rheumatology Unit of Padova University Hospital were enrolled in the study and an equal number of healthy volunteers were recruited as control group. This observational case-control study was carried out from March to June 2018. The inclusion criteria were: (1) patients affected by hereditary periodic fevers (FMF, TRAPS, and MKD); (2) attack-free patients; (3) patients with age ranged from 18 to 70 years old. Exclusion criteria were: (1) secondary causes of osteoporosis such as endocrinologic disorders, renal diseases, and malabsorption syndromes; (2) the usage of drugs that can affect bone mineral density (BMD), such as glucocorticoids, heparin, anticonvulsant drugs, or hormone therapy for breast or prostate cancer; also, patients undertaking bisphosphonates, denosumab, teriparatide, and SERMs were excluded; (3) cognitive impairment; (4) pregnancy and (5) women at breastfeeding time. Thus, we enrolled 31 patients affected by FMF, 6 patients with TRAPS and 3 patients with MKD. The diagnosis of FMF was based on the Tel Hashomer Criteria (6), while TRAPS and MKD were diagnosed according to clinical features, physical examination, and genetic analysis. All patients and controls were questioned on their family history for OP, smoking, and alcohol consumption and history of prior fragility fracture. Patients with SAIDs were also questioned regarding duration of the disease. Fasting blood and urine samples were collected in order to determine Calcium (Ca), Phosphorus (P), Magnesium (Mg), 24-h urine Calcium, 24-h urine Phosphorus, Albumin, Creatinine, C-terminal telopeptide of collagen I (CTX), bone Alkaline Phosphatase (bALP), and Serum Amyloid A (SAA). Calcium corrected for albumin (mmol/l) was calculated as follow: Serum Ca + 0.02 × [40–Albumin (g/l)] (7). Vitamin D [25(OH)D] and parathyroid hormone (PTH) were measured by commercial kits using a chemiluminescent enzyme immunoassay (CLIA). Moreover, aliquots of serum were immediately frozen at −80°C in order to determine serum osteoprotegerin (OPG) and Receptor Activator of Nuclear factor κB Ligand (RANK-L) levels using a commercially available ELISA kit (*Pantec, Turin, Italy)*. Femur and lumbar Dual-energy X-ray Absorptiometry (DXA) scans were performed with QDR Bone Densitometer Discovery (*Hologic Inc., Waltham, MA*). The *in vitro* coefficient of variation, measured by analyzing an anthropomorphic phantom reproducing the lumbar vertebrae ten times, was 0.6%. *In vivo* reproducibility was measured by obtaining five scans in 1 week from ten healthy volunteers; the coefficient of variation (CV) was 1.2% for the lumbar spine, 2.1% for the whole femur, and 1.8% for the femoral neck. The BMD was expressed in g/cm^2^ and standard deviation scores (T and Z scores) were determined. In post-menopausal women and men older than 50 years, osteoporosis is defined when the T-score is below −2.5, osteopenia when the T-score is between −1 and −2.5, and normal BMD when the T-score is higher than −1. In pre-menopausal women and men younger than 50 years, the *Z*-score compares the BMD to a subject of the same age and sex: if it is below −2, it is defined as “below the expected range for age.” The Trabecular Bone Score (TBS) was calculated following the standard analysis procedures recommended by the manufacturer. TBS was calculated on DXA lumbar images, using iNsight Software (*version 1.8.0.0; Medimaps, Merignac, France*). On DXA images, morphometric vertebral fracture assessment (VFA) was obtained in order to identify possible vertebral fractures, according to Genant's criteria (8). All measurements were taken by the same operator. We also estimated the individual fracture risk probability at 10 years by using the fracture risk assessment tool (FRAX^®^) when possible (i.e., in subjects older than 40). All subjects gave their fully written informed consent at enrolment. The study was performed in accordance with the principles of the Declaration of Helsinki and approved by the Local Ethics Committee of the University-Hospital of Padova (Protocol number: 0060237 of 16/11/12).

### Statistical Analysis

The Kolmogorov-Smirnov test was used to test the normal distribution of the data. Results are reported as frequency and proportion for categorical data and as median (maximum; minimum) or mean ± Standard Deviation (SD) for continuous variables. The Mann-Whitney U Test was performed for compared continuous variables and Chi-square test for categorical data. Significance was accepted for values of *p* <0.05. Spearman's Rank Order Correlation coefficient was applied in order to determine the direction of the relationship between BTM and SAA. We used *GraphPad Instat* version 3.00 for all the statistical analysis.

## Results

The anthropometrics and demographic characteristics of the two groups are recorded in Table 1. There is no statistically significant difference between the ages of the groups (*p* = 0.6304). Similarly, no differences were observed in terms of sex, Body Mass Index (BMI), family history of fractures, prior fragility fractures, and common habits (smoke and/or alcohol intake). Two women with SAIDs were in early menopause (defined as menopause before 45), and one woman among the controls. Mean disease duration for patients was 6.025 ± 4.05 years. Concerning the therapy, 19 out of 40 of patients were in treatment with colchicine at the dosage of 1 mg/day and 21 of them were previously in therapy with it. Moreover, 7 patients out of 40 were in therapy with the biologic drug anti IL-1 (Anakinra). Table 2 shows the mutations/polymorphisms carried by our patients, subdivided into each autoinflammatory disease considered. The most frequently observed variant for FMF was R202Q, a polymorphism associated to a mild clinical phenotype and to a high linkage-disequilibrium and pro-inflammatory effect. When homozygosity occurs, there is an increased risk for a severe form of FMF, resulting, albeit rarely, in systemic amyloidosis (9). In TRAPS patients, R92Q was the most frequent low-penetration mutation observed. Interestingly, one patient with MKD presented I268T, a mutation associated mostly to mevalonic aciduria and retinitis pigmentosa, and one presenting V377I, which is considered a fully pathogenetic mutation (*INFEVERS*, www.infevers.umai-montpellier.fr). Concerning phosphocalcic metabolism, as recorded in Table 3, no statistically significant difference was observed between the two groups except for phosphorus levels, which was lower in patients, however, within the normal range. As expected, the main inflammatory marker investigated in our study (SAA) resulted higher in patients affect by SAIDs compared to controls with a statistically significant difference (*p* = 0.0244). Regarding BTM, we found no differences in b-ALP, CTX and RANK-L serum levels, while OPG was significantly increased in affected patients (*p* = 0.0064; Table 3). Lumbar, femur neck and total femur BMD values did not differ between the two groups, and no statistically significant difference was observed for the T and Z scores in all the sites considered. Furthermore, TBS was similar among the two groups with no statistically significant difference (*p* = 0.8947). Analyzing the morphometric VFA DXA images, no vertebral fractures were identified in both groups and no prior fragility fractures are reported in the patient's history. We observed no correlation between SAA and OPG and SAA and b-ALP. However, there is a negative correlation (*r* = −0.48 and *p* = 0.002) between SAA and CTX. An increased FRAX® score for major osteoporotic fractures was detected in SAIDs patients compared to controls, although it is not higher than 20% which is the threshold value recommended by the National Osteoporosis Foundation Guide (10) (Table 4).

**Table 1:** Demographic characteristic of our cohort and controls.

	**Patients, *n* = 40**	**Controls, *n* = 40**
Mean age ± SD (years)	41.52 ± 13	42.90 ± 12.90
Sex (male/female)	9/31	11/29
BMI ± SD (Kg/m^2^)	22.37 ± 3.70	23.25 ± 3.70
Smokers	9	3
Alcohol intake	1	1
Menopausal age before 45 years-old	2	1
Family history of fragility fractures	9	5
Prior fragility fractures	0	0
Vertebral fractures	0	0
Mean disease duration from diagnosis ± SD (years)	6.03 ± 4.05	nd
Therapy	19/40 Currently in therapy with colchicine 21/40 Previously in therapy with colchicine 7/40 In therapy with anti IL-1	–

*BMI, body mass index*.

**Table 2:** Mutations/polymorphisms carried by our patients and subdivided by the three monogenic diseases considered.

	**No mutations (clinical diagnosis or neutral polymorphisms)**	***MEFV* gene (FMF)**	***TNFRSF1A* gene (TRAPS)**	***MVK* gene (MKD)**	**Total**
n. patients	16	15	6	3	40
Variants		**R202Q** (rs224222) **K695R** (rs104895094) **E148Q** (rs3743930) **P369S** (rs11466023)	**R92Q** (rs4149584)	**I268T** (rs104895304) **S52N** (rs7957619) **V377I** (rs28934897)	

*FMF, familial mediterranean fever; TRAPS, TNF-receptor associated periodic syndrome; MKD, mevalonate kinase deficiency. “rs” ID is an identification tag assigned by NCBI that identify uniquely the submitted variant*.

**Table 3:** Phosphocalcic and inflammatory markers serum levels of the two groups.

	**Patients**	**Controls**	***p*-value**
Calcium (mmol/l)	2.40 (2.25–2.64)	2.33 (2.20–2.55)	ns
Albumin (g/l)	47 (38–53)	46.50 (41–52)	ns
Calcium Corrected for Albumin	2.40 (2.25–2.46)	2.29 (2.26–2.39)	ns
Phosphorus (mmol/l)	0.91 (0.65–1.33)	1.01 (0.59–1.5)	0.0196
Magnesium (mmol/l)	0.85 (0.72–0.99)	0.84 (0.72–0.99)	ns
Ca-U24 h (mmol/24 h)	3.69 (0.76–8.86)	3.88 (0.90–10.42)	ns
P-U24 h (mmol/24 h)	23 (8.50–50.50)	24.20 (12.20–51.1)	ns
Creatinine (umol/l)	67.50 (54–101)	70 (53–101)	ns
Vitamin D (nmol/l)	58.50 (12–141)	61 (14–139)	ns
PTH (ng/l)	22.80 (10.70–78.40)	22.75 (8.40–56.90)	ns
b-ALP (μg/l)	9.90 (5.20–19.20)	9.80 (6.20–14.80)	ns
CTX (pg/ml)	224.20 (38–997)	220.25 (33–690.60)	ns
SAA (mg/l)	9.80 (1.30–102)	2.78 (0.82–11.40)	0.0244
RANK-L (pmol/l)	0.01 (0.01–0.25)	0.02 (0.01–0.24)	ns
OPG (pmol/l)	6.56 (1.18–10.50)	3.30 (1.48–8.99)	0.0064

*Data are expressed as median (min–max). Ca-U24 h, 24-h urine Calcium; P-U24 h, 24-h urine Phosphorus; PTH, parathyroid hormone; b-ALP, bone Alkaline Phosphatase; CTX, C-terminal telopeptide of collagen I; SAA, Serum Amyloid A; RANK-L, Receptor Activator of Nuclear factor κB Ligand; OPG, osteoprotegerin; ns, not significant*.

**Table 4:** Values of densitometric examination in patients and controls.

	**Patients**	**Controls**	***p*-value**
Lumbar spine BMD (g/cm^2^)	0.99 (0.77; 1.13)	0.94 (0.68; 1.30)	ns
Lumbar spine *Z*-score	−0.20 (−2.80; +2.20)	−0.15 (−2.10; +4)	ns
Lumbar spine *T*-score	−0.40 (−2.90; +2)	−0.70 (−3.30; +3.10)	ns
Femur neck BMD (g/cm^2^)	0.79 (0.50; 1.14)	0.78 (0.60; 1.11)	ns
Femur neck *Z*-score	−0.30 (−1.80; +2.70)	−0.45 (−1.80; +1.80)	ns
Femur neck *T*-score	−0.50 (−3.10; +2.70)	−0.70 (−2.20; +1.90)	ns
Total femur BMD (g/cm^2^)	0.90 (0.59; 1.18)	0.90 (0.66; 1.12)	ns
Total femur *Z*-score	−0.20 (−1.80; +1.30)	−0.20 (−1.60; +1.60)	ns
Total femur *T*-score	−0.40 (−2.80; +1)	−0.40 (−2.30; +1.10)	ns
TBS L1-14	1.36 (1.15; 1.56)	1.38 (1.09; 1.58)	ns
Morphometric vertebral fractures	0	0	ns
FRAX® for major osteoporotic fractures	2.60 (1.70; 12)	5.10 (2; 11)	0.0139
FRAX® for hip fractures	0.40 (0; 4)	0.15 (0; 2.30)	ns

*Data are expressed as median (min; max). BMD, bone mineral density; TBS, trabecular bone score; ns, not significant*.

## Discussion

At best of our knowledge, only a few studies examined bone metabolism in autoinflammatory diseases. There are few reports on FMF patients (9, 11–14), however there are no evidences of studies concerning bone homeostasis in TRAPS and MKD. Thus, in our work we tried to assess if an impairment of the bone system may develop in patients affected by a different subset of autoinflammatory diseases with similar clinical manifestations and a common inflammatory background. However, the mechanisms by which chronic inflammation acts on bone metabolism remain partially unknown and a full consensus on the possible onset of OP in a context of autoinflammation has not been achieved yet. In our cohort, the results on BTM are consistent with previous reports that observed higher OPG levels in serum of FMF patients (15). This evidence may support the idea that OPG exerts its protective role even in a context of hyper-inflammation. In particular, it seems to reflect a preventive mechanism against bone loss or even a response toward pro-inflammatory cytokines, since high levels of OPG are detected also in other inflammatory pathologies such as intestinal bowel disease (IBD), rheumatoid arthritis (RA) and juvenile idiopathic arthritis (16–18). However, in the majority of the chronic inflammatory and autoimmune diseases osteoporosis may be a frequent comorbidity (19, 20), despite the protective action exerted by OPG. For example RA is the most frequent rheumatological disease presenting a bone impairment (21). In addition, glucocorticoids intake and a general reduced patient mobility, typical of chronic diseases, should be taken into account as secondary causes of OP. Different studies demonstrated that in chronic inflammatory diseases many cytokines such as TNF-alpha, IL-1, IL-6, IL-11, IL-15, and IL-17 are associated to bone resorption and osteoclasts activation (22). Indeed, these cytokines are able to increase the effects of RANK-L through its receptor RANK, one of the most important bone reabsorption factor (3, 4), and to enhance mechanisms of up-regulation of genes activating RANK-L pathway. This is supported also by studies carried out on murine models, that demonstrate a close crosstalk between pro-inflammatory cytokines and bone (23). Interestingly, it was observed that IL-1 may increase RANK-L and MCSF (macrophage colony stimulating factor), upregulate TNF-α and eventually lead to osteoclasts differentiation. In addition, IL-1 may decrease OPG mRNA expression and promotes osteoclasts survival. Similarly, also IL-6 is involved in RANK-L upregulation and osteoblasts suppression (22). Despite this close relationship between pro-inflammatory cytokines and RANK-L, in our cohort we observed that RANK-L serum levels are not significantly different if compared to the control group, highlighting that several players may regulate bone homeostasis in different ways. In this context, it is known that one of the pivotal inflammatory marker suitable for monitoring SAIDs progression and worsening is SAA (24). SAA is an acute phase reactant released into the circulation from the liver in response to infections or injuries and it is able to induce other pro-inflammatory cytokines and chemokines production (25). It is known that even during the asymptomatic attack-free periods, when the subclinical inflammation persists, SAA levels remain high in patients perpetuating possible systemic damages (26). In recent years, some discordant evidence associating SAA to *in vitro* bone remodeling emerged. In a study from Thaler et al. (27) it was hypothesized that SAA may presents pleiotropic roles on bone homeostasis functioning as a paracrine factor, since it is associated with ECM repair, bone remodeling and absorption, osteoblast progression, and skeletal development. Similarly, Oh et al. demonstrated that SAA is able to inhibit RANKL-induced osteoclast formation by downregulating genes typically involved in osteoclast differentiation (28) and other findings supporting these evidences are reported in a work from Kim et al. (29). Furthermore, SSA together with other acute phase reactants can up-regulate Interferon Regulatory Factor 8 (IRF8) mRNA expression which, in turn, suppresses genes, such as *cathepsin K* and *TRAP*, responsible for osteoclasts development. Taken together, these data suggest that, although SAA it is a pro-inflammatory cytokine, a modulatory role directly exerted by SAA on bone homeostasis regulation is conceivable. Although we did not observe a significant correlation between SAA and OPG and SAA and b-ALP, we noticed that there is a negative correlation between CTX and SAA and this may enhance our hypothesis about the osteoprotective role of SAA. In our cohort as expected, we observed higher levels of SAA compared to controls, consistent with the context of inflammation of SAIDs. Thus, the evidence that SAA can potentially determine a positive effect on bone homeostasis by downregulating RANK-L and our finding that OPG is more expressed in SAIDs patients may explain the lack of increased occurrence of osteoporosis in our cohort. This data was also confirmed by the DXA analysis. Indeed, regarding BMD we did not find any difference in all the sites examined according to three other published works (12–14). However, in a previous study by Yildirim et al. carried out on 28 FMF patients a lower BMD in all the bone sites considered was observed (15). We also did not find a significant difference in TBS between patients and controls and far as we know, this is the first study that investigate this parameter in SAIDs. We observed in our patients a slight increase in FRAX® score for major osteoporotic fractures, unrelated to the diseases. No vertebral fractures were indeed identified at the morphometric VFA using DXA images, neither in young patient nor in the older. This supports once again the hypothesis that the fracture risk is not increased in our patients. However, this technique have some limitations such as lower spatial resolution and non-accessibility of the upper thoracic level (30). Concerning bone metabolism, we did not observe any difference compared to the control group. However, our patients had significant lower phosphorus serum levels, nonetheless within the normal range. We observed a comparable 24 h phosphorus urine levels, excluding renal phosphorus loss. The hypophosphatemia may be explained with a decreased intestinal absorption of phosphate or an increased urinary excretion probably due to the therapy with colchicine (31). Moreover, since colchicine is known to prevent OP (13, 32), the bone loss may be, in a certain way, preserved in treated patients, and consequently OP should be less observed. There are some limitations in our study. To prove the modulatory effect on bone homeostasis of SAA, further studies in order to investigate the reduced osteoclast activation would be appropriate. Moreover, the small sample size, particularly of those affected by TRAPS and MKD, needs further researches including a more comprehensive and large cohorts of patients.

## Conclusion

Our data support the evidence that patients affected by SAIDs do not present an increased risk of OP. However, a decrease of BMD in patients may occur despite the prevalence of OP is not higher. The TBS in patients was similar to controls, highlighting a preserved bone quality in our patients. No vertebral fractures were indeed identified. The increased OPG in our cohort may represent a re-balancing factor on bone remodeling, but this evidence needs further investigation. Finally, it should be taken into account a new role for SAA, a pro inflammatory maker able to reduce osteoclast activation by determining a modulatory effect on bone homeostasis

## Data Availability

The raw data supporting the conclusions of this manuscript will be made available by the authors to any qualified researcher.

## Ethics Statement

The studies involving human participants were reviewed and approved by Local Ethics Committee of the University-Hospital of Padova (Protocol Number: 0060237 of 16/11/12). The patients and participants provided their written informed consent to participate in this study.

## Author Contributions

SB recruited patients, analyzed data, prepared tables, designed and wrote the manuscript. GF conceived the concept, recruited patients, wrote the manuscript and participated to the analysis. PG critically evaluated and revised the manuscript. MZ supported laboratory data analysis. VC performed densitometric analysis and revised the manuscript. PS supervised results, revised the manuscript, participated to data analysis and was responsible for its financial supports and the corresponding works. All the authors approved the final manuscript.

### Conflict of Interest Statement

The authors declare that the research was conducted in the absence of any commercial or financial relationships that could be construed as a potential conflict of interest.
